# Post-translational modifications of voltage-gated sodium channels in chronic pain syndromes

**DOI:** 10.3389/fphar.2015.00263

**Published:** 2015-11-05

**Authors:** Cedric J. Laedermann, Hugues Abriel, Isabelle Decosterd

**Affiliations:** ^1^F.M. Kirby Neurobiology Research Center, Boston Children’s Hospital, Harvard Medical School, BostonMA, USA; ^2^Department of Clinical Research, University of BernBern, Switzerland; ^3^Pain Center, Department of Anesthesiology, Lausanne University Hospital (CHUV) and University of LausanneLausanne, Switzerland; ^4^Department of Fundamental Neurosciences, University of LausanneLausanne, Switzerland

**Keywords:** voltage-gated sodium channels, post-translational modification, chronic pain, hyperexcitability, nociceptive neurons

## Abstract

In the peripheral sensory nervous system the neuronal expression of voltage-gated sodium channels (Na_v_s) is very important for the transmission of nociceptive information since they give rise to the upstroke of the action potential (AP). Na_v_s are composed of nine different isoforms with distinct biophysical properties. Studying the mutations associated with the increase or absence of pain sensitivity in humans, as well as other expression studies, have highlighted Na_v_1.7, Na_v_1.8, and Na_v_1.9 as being the most important contributors to the control of nociceptive neuronal electrogenesis. Modulating their expression and/or function can impact the shape of the AP and consequently modify nociceptive transmission, a process that is observed in persistent pain conditions. Post-translational modification (PTM) of Na_v_s is a well-known process that modifies their expression and function. In chronic pain syndromes, the release of inflammatory molecules into the direct environment of dorsal root ganglia (DRG) sensory neurons leads to an abnormal activation of enzymes that induce Na_v_s PTM. The addition of small molecules, i.e., peptides, phosphoryl groups, ubiquitin moieties and/or carbohydrates, can modify the function of Na_v_s in two different ways: via direct physical interference with Na_v_ gating, or via the control of Na_v_ trafficking. Both mechanisms have a profound impact on neuronal excitability. In this review we will discuss the role of Protein Kinase A, B, and C, Mitogen Activated Protein Kinases and Ca++/Calmodulin-dependent Kinase II in peripheral chronic pain syndromes. We will also discuss more recent findings that the ubiquitination of Na_v_1.7 by Nedd4-2 and the effect of methylglyoxal on Na_v_1.8 are also implicated in the development of experimental neuropathic pain. We will address the potential roles of other PTMs in chronic pain and highlight the need for further investigation of PTMs of Na_v_s in order to develop new pharmacological tools to alleviate pain.

## Pain, NOCICEPTION, INFLAMMATORY AND NEUROPATHIC PAIN

The ability to recognize and remember danger is one of the major evolutionary steps necessary for survival in a hostile environment. The primary line of defense for organisms relies on the primary nociceptive neurons, which are activated when stimuli intensities reach the noxious range. The peripheral endings of nociceptive neurons are found in almost all tissue of the body, i.e., skin, muscle and internal organs (Kruger et al., 2003). Their cell bodies are located in the dorsal root ganglia (DRG) or the trigeminal ganglia (TG) for the innervations of the face. Central terminals of primary nociceptive neurons project to the dorsal horn of the spinal cord where they make their first synapse with secondary order sensory neurons and/or interneurons (Millan, 1999). At this level, the transmission of the signal by the secondary order projection neurons to the supra spinal centers is modulated by a complex network of the dorsal horn. A barrage of activity or neurotransmitter release from the periphery can highly modulate the excitability of the secondary order neurons (Latremoliere and Woolf, 2009). Spinal neurons are subjected to change by microglial, astrocytic and oligodendrocitic cells (Fotia et al., 2002; Lou et al., 2005). Furthermore, descending projections from supra-spinal centers can affect the central terminal of the primary sensory neuron, the projection neuron and the inhibitory and excitatory interneurons. The signal is eventually processed and reaches supra-spinal centers, including brain areas involved in sensory and emotional pain perception. At this point, complex circuitries process and integrate the pain signal and allow for appropriate behavioral and motor responses. These circuitries also modulate pain signaling via descending pathways that ultimately reach the dorsal horn neurons.

For the most part, primary nociceptive neurons are polymodal (Dubin and Patapoutian, 2010), in that they are able to detect a wide range of stimuli, such as heat and mechanical or chemical stimuli. To integrate these wide ranges of stimuli, nociceptive neurons express a multitude of receptors and ion channels in their free endings (Basbaum et al., 2009). There are many review articles that extensively discuss these so called transducers, i.e., TRP channel family members or acid-sensing ion channels (ASICs; Basbaum et al., 2009; Stucky et al., 2009; Deval et al., 2010). Once transducers are activated, these non-selective ion channels open and contribute to cell depolarization, thus eliciting an action potential (AP) and allowing for the transmission of a noxious signal along pain pathways.

Nociception enables the organism to react quickly, and by virtue of the emotionally offensive component of pain, helps the organism avoid similar situations in the future. When the noxious stimulus is acute, pain is transient and the nociceptive neurons should subsequently return to a resting state. This process is referred to as **nociception**. On the other hand, tissue injury leads to longer lasting inflammatory pain, characterized by peripheral sensitization. **Inflammatory pain** is due to the modification of the chemical environment surrounding nociceptive neurons and the accumulation of several factors secreted by recruited non-neural cells, such as mast cells, macrophages, neutrophils, inflammatory cells, fibroblasts and keratinocytes, as well as by the nociceptive neurons themselves. These factors are of diverse origins but include protons (H^+^), nerve growth factors (NGFs), cytokines (such as IL-1β, IL-6), tumor necrosis factor alpha (TNF-α), prostaglandins (PGE_2_), several neurotransmitters (serotonin, ATP) and peptides (bradykinin, substance P, CGRP). This mixture is commonly referred to as “the inflammatory soup” (Basbaum et al., 2009), and will increase spontaneous neuronal firing, usually decrease the threshold of nociceptive neurons and increase firing in response to suprathreshold stimuli. The mechanisms by which inflammatory pain increases pain transmission include the activation of kinases that phosphorylate membrane channels and receptors, which subsequently alter their function, and the genetic regulation of primary sensory neurons (Woolf and Costigan, 1999). Inflammatory pain, by the virtue of central sensitization, is also accompanied by the local loss of inhibition (Julius and Basbaum, 2001) and enhanced postsynaptic transmission (Galan et al., 2004).

Inflammatory pain is linked to the persistence of inflammation, but should fade away when the tissue is healed. In some cases, however, abnormal activity from the peripheral neurons can also occur in the absence of tissue inflammation. This abnormal activity occurs when nociceptive neurons are damaged and elicit long-term molecular modifications that eventually lead to **neuropathic pain**. Neuropathic pain is defined as the “pain caused by a lesion or disease of the somatosensory system” (Heikamp et al., 2014). This definition reflects that not only nerve injury, but also degenerative, infectious or metabolic conditions can lead to neuropathic pain, accounting for the distinct etiologies of peripheral neuropathic pain (e.g., compressive disk herniation, diabetic neuropathy, chemotherapeutic-induced neuropathy, post-herpetic neuralgia, etc.) (Woolf and Mannion, 1999). The prevalence of neuropathic pain varies from 7 (Bouhassira et al., 2008) to 18% of the population (Toth et al., 2009). Most of these patients are often resistant to treatment (Brower, 2000). Neuropathic pain cardinal positive symptoms are spontaneous pain, allodynia and hyperalgesia, but can be also associated with negative symptoms such as hyposensitivity in a related nerve territory. Spontaneous pain is thought to arise from ectopic activity, which can be driven by C- (Djouhri et al., 2006) and A-fibers (Liu et al., 2000); where as allodynia and hyperalgesia are related to reduced activation thresholds or an increased response of primary afferent neurons (von Hehn et al., 2012). The peripheral mechanisms underlying neuropathic pain associated hyperexcitability have been extensively reviewed in other articles (Julius and Basbaum, 2001; Woolf, 2004; Campbell and Meyer, 2006; Hucho and Levine, 2007). These mechanisms include altered gene expression, dysregulation of membrane channel expression, migration of inflammatory cells and activation of satellite cells in DRG neurons. The increased peripheral input leads to activity-dependent mechanisms of central sensitization in the spinal cord and supra-spinal levels (Woolf and Salter, 2000; Latremoliere and Woolf, 2009; Woolf, 2011). Although they share similar pain symptoms and some common mechanisms, inflammatory and neuropathic pain differ fundamentally by their respective pharmacology and resolution. Altogether, peripheral and central mechanisms will lead to enhanced and long-lasting pain perception, which will ultimately result in debilitating chronic conditions, sleep disturbances, depression, anxiety and social withdrawal (Bair et al., 2003; Turk et al., 2010). On the other side of the spectrum, in diseases where patients can no longer experience pain appropriately, such as in congenital insensitivity to pain, the pain signal is permanently shut off. This results in an inappropriate behavioral response and a reduced life expectancy in a subset of individuals (Nagasako et al., 2003).

## Na_v_s: Structure And Function

After activation via transducers, primary nociceptive neurons transmit the signal along the axon to the spinal cord. Voltage-gated sodium channels (Na_v_s) play a critical role in this process. Na_v_s are activated upon depolarization of the transmembrane voltage, generating a fast, transient and massive inward sodium current, which accounts for the rising phase of the AP. Na_v_s give rise to the upstroke of the AP and contribute to setting the resting membrane potential of nociceptive neurons (Herzog et al., 2001; Rush et al., 2007).

Since the first biochemical characterization of Na_v_s (Beneski and Catterall, 1980), tremendous effort has been made to unravel the structure and function of the sodium channel. The α-subunit is encoded by a single gene, which is structurally divided into four homologous domains (I-IV) connected by an intra and/or extracellular loop, thus referred to as heterotetramere. Each domain is composed of six α-helical transmembrane segments. S5 and S6 compose the pore of the channel, whereas S1 – S4 are the voltage sensors. The crystallography structure of Na_v_Ab, a bacterial sodium channel from *Arcobacter butzleri*, was identified in 2011 (Payandeh et al., 2011). Although Na_v_Ab is a homotetramere and the mammalian Na_v_s is heterotetrameric, they share similar pharmacological profiles (Ren et al., 2001). Most of the inferences made based on previous biochemical and electrophysiological experiments have been confirmed (Payandeh et al., 2011).

Nine discrete genes (*SCNxA*) encode for the α-subunits (Na_v_1.1 to Na_v_1.9 isoforms) (Catterall et al., 2005) and another atypical tenth isoform, NaX (Akopian et al., 1997; Noda and Hiyama, 2014). Each isoform has its own biophysical properties and particular expression pattern across the nervous system. A single amino acid substitution in the S5–S6 linker renders Na_v_1.5, Na_v_1.8, and Na_v_1.9 resistance to TTX.

α-subunits are accompanied by associated β-subunits with an assumed stoichiometry for an α-β association of 1:1 (Catterall, 1992). There are four different identified genes coding for the different β-subunits: *SCN1B* codes for β1 (Isom et al., 1992) and its associated splice variant β1A (Kazen-Gillespie et al., 2000); *SCN2B* codes for β2 (Isom et al., 1995a); *SCN3B* codes for β3 (Morgan et al., 2000) and *SCN4B* codes for β4 (Yu et al., 2003). The pore-forming α-subunit enables for Na^+^ conductance, but the β–subunits can modulate the biophysical properties and plasma membrane stabilization of Na_v_s (Isom et al., 1995b).

## Na_v_1.7, Na_v_1.8, and Na_v_1.9 Are Expressed In Nociceptive Neurons

Na_v_s are broadly expressed in excitable cells throughout the body, with some isoforms ubiquitously expressed and others expressed in specific tissues. In nociceptive neurons, many of the different Na_v_s isoforms are present and collaborate with one another for electrogenesis. With the exception of Na_v_1.2 and Na_v_1.4, all the Na_v_ isoforms are expressed in DRG nociceptive neurons (Black et al., 1996; Rush et al., 2007; Berta et al., 2008; Fukuoka et al., 2008; Fukuoka and Noguchi, 2011; Ho and O’Leary, 2011). The role of Na_v_1.5 in adult small DRG neurons has not been fully unraveled (Renganathan et al., 2002). As compared to Na_v_1.1 and Na_v_1.6, the Na_v_1.7 isoform is the most expressed TTX-sensitive isoform among the DRG neurons (Black et al., 1996; Toledo-Aral et al., 1997; Berta et al., 2008; Ho and O’Leary, 2011; Dib-Hajj et al., 2013). The two TTX-resistant isoforms, Na_v_1.8 and Na_v_1.9, are also highly expressed in nociceptive neurons (Akopian et al., 1996; Dib-Hajj et al., 1998). During the last decade, mutations in Na_v_1.7, Na_v_1.8, and Na_v_1.9 have been linked with human pain disorders (see Inherited Pain Syndromes). The possible variable combinations of each of these “pain specialized” isoforms, as well as their relative expression levels, differentially shape the AP and firing properties, accounting for the heterogeneity among DRG neurons (Rush et al., 2007; Theriault and Chahine, 2014). In this review, we focus on the roles of Na_v_1.7, Na_v_1.8, and Na_v_1.9 in chronic pain states.

## Na_v_s’ Implication In Pain Syndromes

Reviewing all the studies that have investigated the Na_v_1.7, Na_v_1.8, and Na_v_1.9 mutations associated with painful channelopathies and those studies that have investigated the expression of Na_v_s in human and animal models of pathological pain is beyond the scope of this review and can already be found in other recent reviews (Brouwer et al., 2014; Waxman et al., 2014; Waxman and Zamponi, 2014; Hoeijmakers et al., 2015). In the next two chapters we will summarize some of the key findings demonstrating the role of Na_v_s in channelopathies and in animal models of inflammatory pain, and we will discuss conflicting results observed in neuropathic pain in both human and animal studies.

### Inherited Pain Syndromes

The contribution of Na_v_1.7, Na_v_1.8, and Na_v_1.9 in chronic pain syndromes is exemplified through human mutations (familial and *de novo* mutations) of these channels and their associated pathologies, being either pain hypersensitivity or congenital insensitivity to pain (Dib-Hajj et al., 2009; Liu and Wood, 2011; Waxman et al., 2014). Seminal studies have linked Na_v_1.7 to altered pain sensitivity. A gain of function for this gene leads to inherited painful channelopathies, such as erythromelalgia (Cummins et al., 2004; Yang et al., 2004) and paroxysmal extreme pain disorder (Fertleman et al., 2006). Conversely, a loss of function for Na_v_1.7 was reported to be associated with congenital insensitivity to pain (Cox et al., 2006). Many other studies have identified Na_v_1.7 mutations as being implicated in numerous altered pain sensation pathologies (Dib-Hajj et al., 2005, 2013; Waxman and Dib-Hajj, 2005; Fertleman et al., 2006; Novella et al., 2007; Cheng et al., 2008). A gain of function mutation of Na_v_1.9 was linked to an episodic pain disorder (Zhang et al., 2013); while another gain of function mutation of Na_v_1.9 was reported to cause a loss of pain perception (Leipold et al., 2013). In the latter the authors showed that the excessive activity of Na_v_1.9 at resting voltages caused sustained depolarization of nociceptive neurons, leading to the inactivation of other Na_v_s and subsequently to the impairment of the AP generation. Other studies also highlighted a role of Na_v_1.7 in idiopathic small-fiber neuropathy (I-SFN); nearly 30% of patients suffering from this pathology had a gain of function mutation in Na_v_1.7 (Faber et al., 2012a). Small fiber peripheral neuropathy is a type of peripheral neuropathy that occurs from damage to C-fibers and A-δ fibers, which can often lead to exaggerated pain sensitivity (Hoeijmakers et al., 2012). Since these first studies, similar gain of function mutations in Na_v_1.8 and Na_v_1.9 have been reported in I-SFN (Faber et al., 2012b; Han et al., 2014; Huang et al., 2014). None of the previous gain of function mutations of Na_v_1.7, Na_v_1.8, and Na_v_1.9 have been studied in animal models of chronic pain.

### Acquired Pain Syndromes

Apart from intrinsic modifications of Na_v_ channel function, a modification of expression levels will also impact neuronal excitability. Most of the studies that are discussed here investigated Na_v_ expression while assuming that an increased sodium channel expression and conductance would cause neuronal hyperexcitability, something already demonstrated by computer simulations (Matzner and Devor, 1992). However, the link between increased sodium channel expression and hyperexcitability is likely more complex. For instance, computational studies revealed that increasing the sodium conductance might actually decrease the firing rate of neurons (Kispersky et al., 2012). In addition, a gain of function mutation of Na_v_1.9 was recently shown to be associated with congenital insensitivity to pain (Leipold et al., 2013).

Modifications in Na_v_1.7, Na_v_1.8, and Na_v_1.9 expression have been observed in several chronic pain syndromes. Both human studies and experimental pain model studies have helped unravel the role of these isoforms in chronic pain syndromes, including both inflammatory and neuropathic pain.

There is substantial evidence linking Na_v_1.7 to inflammatory pain in animal studies. Studies have reported an increase of Na_v_1.7 expression after injection of pro-inflammatory mediators (Gould et al., 2000; Black et al., 2004). Knocking-down Na_v_1.7 in a model of inflammatory pain with a viral vector in primary afferents (Yeomans et al., 2005) led to the attenuated development of hyperalgesia, a result confirmed by another study using Na_v_1.7 knockout mice (Nassar et al., 2004). Similarly to Na_v_1.7, knocking down (Khasar et al., 1998) or knocking out Na_v_1.8 (Khasar et al., 1998; Akopian et al., 1999) prevented full development of pain hypersensitivity in inflammatory pain models. Na_v_1.8 was also shown to be increased in inflammatory pain models (Coggeshall et al., 2004; Strickland et al., 2008), and its role was confirmed in knockdown studies (Yu et al., 2011) and in studies using specific blockers (Jarvis et al., 2007; Moon et al., 2012). Other studies also support a role for Na_v_1.9 in inflammatory pain (Dib-Hajj et al., 2010). Na_v_1.9 knockout mice or knockdown rats have a weaker response to inflammatory mediator application (Hirade et al., 1999; Priest et al., 2005; Amaya et al., 2006; Lolignier et al., 2011; Hockley et al., 2014).

Dysregulated Na_v_ expression, by altering the intrinsic electrical properties of neuronal plasma membranes, is largely accountable for neuropathic pain-associated hyperexcitability (Matzner and Devor, 1994; Zhang et al., 1997). Na_v_s’ contribution to neuropathic pain is also demonstrated by the application of local anesthetics known to block sodium channels, which suppresses ectopic discharges and attenuates allodynia and hyperalgesia (Mao and Chen, 2000; Suter et al., 2003; Scholz et al., 2005).

The development of nerve injury-induced neuropathic pain animal models has significantly contributed to the discovery of mechanisms that contribute to neuropathic pain syndromes, but also gave rise to conflicting results. For instance, the role of Na_v_1.7 in neuropathic pain is still being debated. Na_v_1.7 mRNA is reduced after peripheral nerve injury-induced neuropathic pain in rats (Berta et al., 2008; Laedermann et al., 2014b; Casals-Diaz et al., 2015), an observation confirmed by reduced levels of the Na_v_1.7 protein. Furthermore, Na_v_1.7 knockout mice still develop neuropathic pain-mediated mechanical allodynia (Nassar et al., 2004). More recent studies suggest that Na_v_1.7 is actually implicated in neuropathic pain by virtue of its concomitant expression in both sympathetic ganglion neurons and nociceptive neurons, rather than solely in nociceptive neurons (Minett et al., 2012). In contrast, other studies have reported an increased expression of Na_v_1.7 mRNA in DRG neurons (Liu et al., 2012), as well as an increased protein expression in the sciatic nerve of animal experimental neuropathic pain models (Laedermann et al., 2013a). In humans, many studies reported an increased expression of this isoform, such as in intervertebral disk injury (Sadamasu et al., 2014), human dental pulp neuromas (Luo et al., 2008b) and other neuromas (Coward et al., 2001a; Kretschmer et al., 2002; Bird et al., 2007; Persson et al., 2011).

Na_v_1.8 regulation is also controversial in experimental nerve-injury induced neuropathic pain. Many studies have reported a downregulation of Na_v_1.8 mRNA (Waxman, 1999; Berta et al., 2008), protein (Decosterd et al., 2002) and currents (Cummins and Waxman, 1997; Berta et al., 2008) in rodent models. Another group, however, reported an increase of the Na_v_1.8-mediated current (Abdulla and Smith, 2002). To reconcile these contradictory findings, it was suggested that a decrease in the expression of Na_v_1.8 mRNA and protein in the cell soma of nociceptive neurons could be due to a redistribution of this isoform in the sciatic nerve (Gold et al., 2003; Thakor et al., 2009). Controlling Na_v_1.8 expression in mice also leads to controversial results. Gene knockout studies did not find a role for Na_v_1.8 in neuropathic pain development (Akopian et al., 1999; Kerr et al., 2001; Abrahamsen et al., 2008), whereas gene knockdown speaks in favor of such a role (Lai et al., 2002; Dong et al., 2007; Leo et al., 2010). It is likely that Na_v_1.8 involvement depends on the type of lesion and the model of chronic pain (Joshi et al., 2006). A few studies carried out in humans showed that Na_v_1.8 expression was increased in neuromas (Kretschmer et al., 2002; Black et al., 2008; Bird et al., 2013).

Na_v_1.9 implication in neuropathic pain has been scarcely investigated. A few studies reported a downregulation of Na_v_1.9 mRNA and protein in animal models of neuropathic pain (Dib-Hajj et al., 1998; Decosterd et al., 2002; Berta et al., 2008; Laedermann et al., 2014b; Casals-Diaz et al., 2015; Yin et al., 2015). A human study reported no modification of Na_v_1.9 protein in patients with lingual nerve neuromas (Bird et al., 2013).

The conflicting observations of sodium channel regulation in animal models of neuropathic pain underscores that further research is necessary to clarify the mechanisms leading to Na_v_s dysregulation and to those that generate hyperexcitability. Some of the aforementioned discrepant results can be attributed to the use of different models of pathological pain, the different species used, the relocalization of mRNA or protein, and/or various compensation mechanisms. Some of these studies analyzed the total pool of cellular Na_v_s, but only Na_v_s anchored at the membrane regulate the electrogenesis of nociceptive neurons. There is a large pool of intracellular Na_v_s in the trafficking pathway (Schmidt et al., 1985), and it is possible that a modification of the membrane fraction can be overshadowed if one looks at the overall cellular pool of sodium channels. Furthermore, it remains possible, that an apparent decrease of total Na_v_ expression in a cell is concomitant with an increase membrane expression of sodium channels. Studying mechanisms that regulate the trafficking of a channel, or mechanisms that directly alter the biophysical properties of a channel, might reconcile these discrepant results.

## Post Translational Modifications

Both acquired and inherited pain syndromes are manifestations of altered function and expression of Na_v_s that result in electrical instabilities in the nociceptive pathway, ultimately leading to pathological pain. In channelopathies, the pain syndrome is due to a DNA mutation that can be either sporadic or inherited. In acquired pain syndromes, the altered expression and function can be due to a plethora of signaling pathway activations. Among them, post-translational modifications (PTMs) are important contributors to the development of chronic pain syndromes.

Post-translational modifications are protein modifications that occur either soon after the ribosome-mediated translation of the mRNA into a polypeptide chain or later in the secretory pathway. These are critical steps for protein maturation and function. In these processes, many different enzymes attach biochemical groups (acetylation, phosphorylation), polypeptides (ubiquitylation, SUMOylation) and complex molecules (glycosylation, isoprenylation), or cleave (proteolysis) a protein’s specific amino acid. The overall effect of PTMs leads to a modulation of the structure, function or localization of the given protein. PTMs were first identified in the study of kinases (Hunter, 2009) and protein degradation (Ciechanover, 2005; Kresge et al., 2006) many decades ago. Since then the number of different PTMs has risen to over 200 (Mann and Jensen, 2003). PTMs are involved in almost every cellular event, from precise gene expression regulation to broad signal integration (Deribe et al., 2010).

Post-translational modifications have a large spectrum of action on proteins, ranging from very stable modifications to very transient and reversible changes. For instance, glycosylation and disulfide bridge formation are directly implicated in the synthesis, maturation and folding of the protein. The covalent binding of molecules, such as the addition of a ubiquitin moiety, leads to quicker and stable protein modification. On the other side of the spectrum, some PTMs are versatile and are important for transient cellular signaling, as exemplified by the phosphorylation process.

There is precise coupling between the interaction sites of PTMs and a given amino acid sequence on the target protein, rendering the system very specific. This also allows for good spatial (many different amino acids can be targeted by the same PTM) and temporal (a given amino acid can be modified by different PTMs) control and allows neurons to fine tune the properties of a protein depending on the changes occurring in the direct environment.

## Ptms Alter Na_v_ Function And/Or Expression

Computational studies have shown that, depending on the site of phosphorylation, the addition of a phosphoryl group that carries two negative charges at physiological pH (Narayanan and Jacobson, 2009) can modify the structure and the function of the protein through an alteration of the free energy landscape. Na_v_s possesses charged residues in the voltage sensor domain that can sense membrane potential oscillation. When the transmembrane voltage changes, these charged domains reorient in the electric field resulting in conformational changes, a process referred to as gating. The addition of charged groups on their intra-cellular, extra-cellular or transmembrane domains modifies protein intrinsic properties and functions. Apart from the direct electrostatic effect on gating of the channels, phosphorylation can also create or disrupt binding sites for interaction with other regulatory proteins that modulate Na_v_ function.

Na_v_s need to be at the interface between a high extracellular and low intracellular sodium concentration to open and drive sodium influx. For this reason they are only functional when anchored at the plasma membrane. There is also, however, a large intracellular pool of Na_v_s in the secretory pathway of the cell (Schmidt et al., 1985; Ritchie et al., 1990). A tight balance between the membrane and the intracellular pool, a process referred to as trafficking, is crucial for the fine-tuning of cellular excitability in nociceptive neurons. Maintaining this equilibrium is largely mediated by PTMs that regulate the trafficking of Na_v_s (Cusdin et al., 2008). Some enzymes will generate PTMs that are responsible for internalization and/or degradation, whereas others will promote externalization or stabilization of Na_v_s at the membrane.

## Peripheral Sensitization Triggers Ptms

We previously highlighted that peripheral sensitization is triggered by the inflammatory soup (Basbaum et al., 2009). There is a large body of evidence that there is a recruitment of macrophages (Perry et al., 1987) and neutrophils (Daemen et al., 1998), as well as degranulation of mast cells (Olsson, 1967; Zochodne et al., 1994) in inflammatory processes, such as the ones observed after nerve injury. Once recruited, these cells secrete peptides, such as prostaglandins (PGE_2_), bradykinin, NGF and serotonin (Cesare et al., 1999; Petho and Reeh, 2012). They also secret cytokines, such as TNF-α or IL-1β and IL-6 (Wells et al., 1992). Some of these mediators are not only secreted by immune or inflammatory cells, but also by the nociceptive neurons themselves (Daemen et al., 1998). When inflammation resolves, these molecules are slowly cleared from the extracellular liquid around nociceptive neurons. In some cases, however, pro-nociceptive molecules, such as NGF and cytokines, (Leung and Cahill, 2010; Dogrul et al., 2011; Gaudet et al., 2011) are found to persist at the site of injury. This phenomenon may partially explain any long-term changes.

**FIGURE 1:**
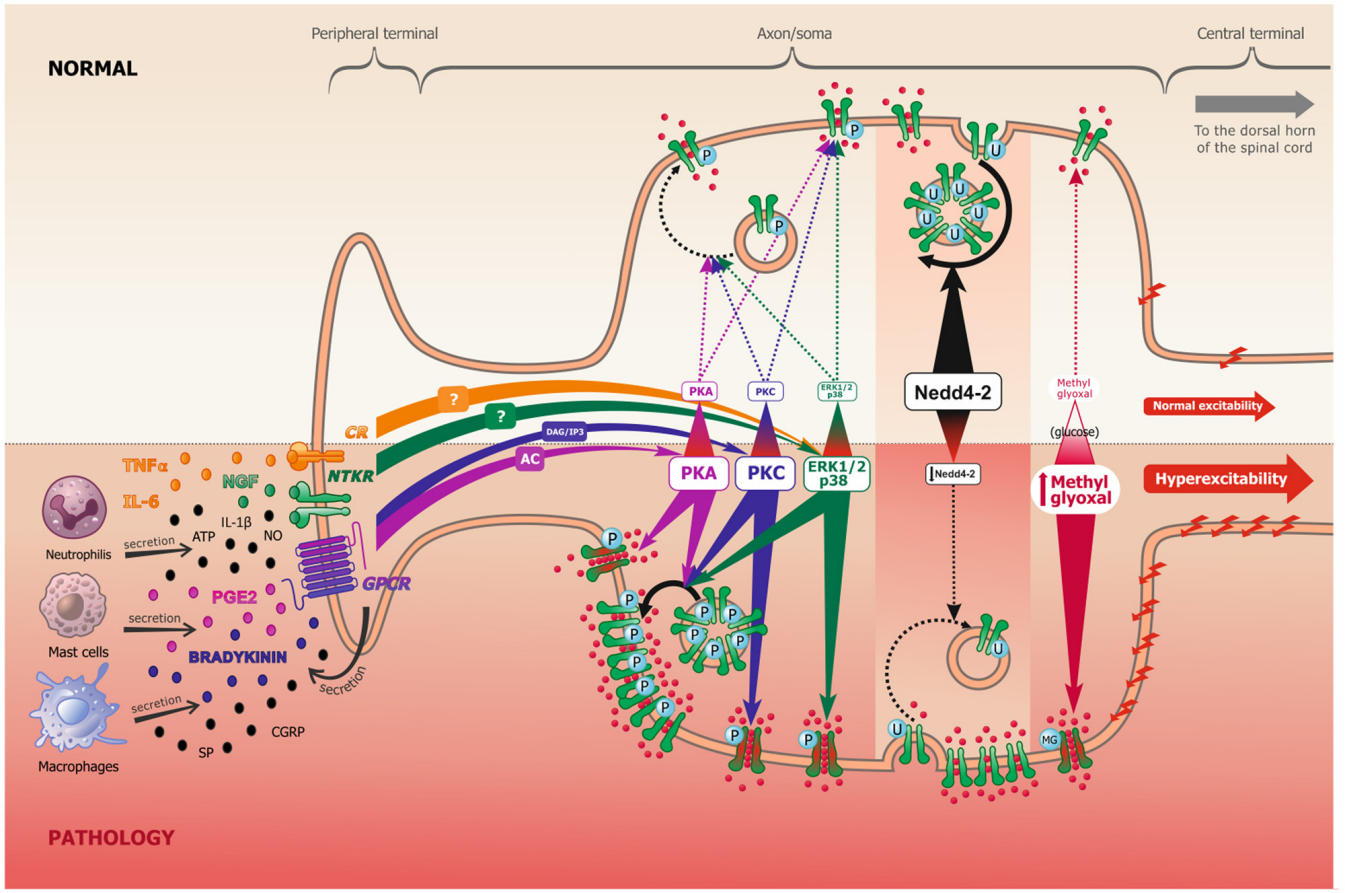
**Scheme representing the peripheral terminal, axon and cell soma of a pseudo-unipolar dorsal root ganglia nociceptive neuron, in normal and pathological pain conditions.** Following tissue damage and inflammation, recruited inflammatory cells secrete numerous pro-inflammatory molecules (referred to as the inflammatory soup). These chemical mediators activate many membrane proteins, including G-protein coupled receptors (GPCR), neurotrophic tyrosine kinase receptors (NTKR) and cytokines receptors (CR). GPCR binding by PGE2 and bradykinin mostly leads to protein kinase A (PKA) and protein kinase C (PKC) activation, through the adenylate cyclase (AC) and inositol 3-phosphate (IP3) secondary messengers, respectively. NTKR and CR binding by TNFα, IL-6 and NGF will activate ERK1/2 and p38 kinases via different potential secondary messengers. These signal transduction pathways can undergo cross communication one with another. In experimental neuropathic pain, nerve injury induces Nedd4-2 downregulation. In models of diabetic neuropathy, as well as in patients, methylglyoxal accumulation is related to an increased glucose concentration. The increased activation of kinases, the downregulation of Nedd4-2 and the accumulation of methylglyoxal all lead to an increase of Na_v_1.7/Na_v_1.8 function (shift of biophysical properties, making the channel more available, as represented in green and red in the figure) and/or to an increase of Na_v_1.7/Na_v_1.8 expression at the membrane (altered trafficking), which leads to increased sodium influx and consequently to nociceptive neuronal hyperexcitability.

When binding to receptors, which include neurotrophic tyrosine kinase receptors (NTKR), such as TrkA, and/or G-protein coupled receptors (GPCRs), such as bradykinin and PGE2 receptors, these aforementioned ligands (peptides and cytokines) will activate multiple intracellular pathways, including Protein Kinase A (PKA), Protein Kinase B (PKB), Protein Kinase C (PKC), Mitogen-activated protein Kinase (MAPK) and the Ca++/Calmodulin-dependent Kinase I and II (CamKI/II), among others (**Figure 1**). The concrete, but not sole, effect of these cascades is phosphorylation or other PTMs of Na_v_s (Dib-Hajj et al., 2010), which lead to long-term increased neuronal excitability. It should, however, be noted that PTMs also control other ion channels, such as potassium channels, and even modulate the activation of some transcription factors. They might also regulate nociceptive neuronal excitability through these alternative mechanisms.

## Protein Kinases

Phosphorylation is an important PTM that affects up to 30% of proteins *in vivo* (Kreegipuu et al., 1999). The phosphate group is usually added to serine, threonine, histidine, and tyrosine residues in eukaryotic proteins. The effect of protein kinases (PKs) on Na_v_s in peripheral chronic pain syndromes has been extensively reported in the literature. The reports are varied and complex, largely due to the important number of phosphorylation sites and to the large number of different PKs present in nociceptive neurons. PKs can modulate Na_v_ function in a very short-term range upon electrostatic interferences. Phosphate groups carry a -2 charge at physiological pH that might interfere with the Na_v_s voltage-sensing domain, or other domains implicated in the gating of the channel. PKs can also have long-term effects when regulating Na_v_ trafficking, which is acheived by masking or highlighting targeting sequences, such as the endoplasmic reticulum (RE) retention signal of Na_v_s (Zhou et al., 2002).

### Protein Kinase A

The immediate increase in nociceptive neuronal excitability observed after tissue injury or inflammation results from G-protein-coupled receptor activation and the resulting intracellular signaling pathway (Woolf and Costigan, 1999). G-protein binding leads to an increase in cAMP production by adenylate cyclase (AC), which ultimately activates PKA. Triggering of this pathway by direct cAMP application or activation of AC is sufficient to elicit hyperalgesia in animals (Taiwo et al., 1989; Kress et al., 1996; Hucho and Levine, 2007). PKA activation not only plays a role in the initiation of pain hypersensitivity, but is also important for the maintenance of inflammatory pain (Aley and Levine, 1999). PGE_2_ is one of the best-known ligands that activates this pathway. PGE_2_ binds to the prostaglandin E_2_ receptor (E_2_) and increases cAMP in sensory neurons (Pierre et al., 2009). Many studies have reported that PGE_2_-induced hyperalgesia is due to PKA activation (Pitchford and Levine, 1991; Khasar et al., 1995; England et al., 1996).

Protein kinase A phosphorylates Na_v_1.2 in brain neurons (Costa and Catterall, 1984a; Rossie and Catterall, 1987), with the main effect being a 50% reduction in the peak current (Gershon et al., 1992), along with modifications of some biophysical properties (Li et al., 1992; Smith and Goldin, 1996). This suggests that both the function and expression of Na_v_1.2 are modified by PKA. Since these early studies, other groups have reported similar decreases in Na_v_1.1, Na_v_1.6, and Na_v_1.7 in different cell expression systems and intact cells (Gershon et al., 1992; Cantrell et al., 1997; Smith and Goldin, 1998; Zhou et al., 2000; Vijayaragavan et al., 2004a; Chen et al., 2008; Liu and Zheng, 2013). The phosphorylation sites of Na_v_1.2 by PKA were mostly investigated using traditional biochemical approaches (Murphy et al., 1993; Smith and Goldin, 1996; Cantrell et al., 1997). A lot of new predictive bioinformatic tools have helped identify phosphorylation sites on other isoforms.

Protein kinase A is increased after inflammation and in pathological pain states. Vijayaragavan et al. (2004a) showed that PKA decreases Na_v_1.7. One could hypothesize, therefore, that inflammation may decrease nociceptive neuronal excitability through Na_v_ downregulation. However, Na_v_1.7, as well as other Na_v_ isoforms, can undergo alternative splicing, with each splice variant possessing distinct biochemical and pharmacological properties (Schaller et al., 1992; Plummer et al., 1997; Dietrich et al., 1998; Schirmeyer et al., 2014). It was previously shown that a particular Na_v_1.7 splice variant, Na_v_1.7 11S, was upregulated and responsible for pain hypersensitivity in animal models of neuropathic pain (Raymond et al., 2004). Contrary to the lack of effect of PKA on three spice variants, the Na_v_1.7 11S splice variant activation curves were shifted to hyperpolarized potentials upon PKA activation, thus lowering the threshold for opening of the channel and presumably increasing neuronal excitability (Chatelier et al., 2008). The current hypothesis is that in chronic pain syndromes the increase of this splice variant, together with the modification of its biophysical properties by PKA, will lead to an increase in sodium conductance which results in nociceptive neuronal hyperexcitability. One must keep in mind that the effect of PKA on Na_v_1.7 was found in *Xenopus* oocytes and other mammalian cell lines, which have a different cellular background than nociceptive neurons, and that Na_v_ regulation varies greatly depending on cell type (Cummins et al., 2001). This is highlighted by one Na_v_1.7 mutation that renders DRG sensory neurons hyperexcitable, but decreases sympathetic DRG neuron excitability (Rush et al., 2006). Studying an endogenous Na_v_1.7 current in adrenal chromaffin cells revealed that cAMP upregulates Na_v_1.7 (Yuhi et al., 1996). Thus, a careful characterization of PKA’s effect on the Na_v_1.7 current in nociceptive neurons needs to be performed.

The effect of PKA on TTX-resistant isoforms is the opposite to that aforementioned. PKA increases the TTX-resistant current (Gold et al., 1996, 1998) in DRG neurons, an effect at least partially due to PGE_2_ (England et al., 1996). A similar increase in the Na_v_1.8 current isolated from a recombinant protein expressed in *Xenopus* oocytes (Vijayaragavan et al., 2004a) and mammalian cells (Fitzgerald et al., 1999) was also reported, providing evidence that PKA activation in pathological pain states can generate nociceptive hyperexcitability through enhanced Na_v_1.8 expression and function. How PKA increases the Na_v_1.8 current is partially due to increased membrane trafficking, since blocking protein transports in DRG neurons prevented a PKA-induced increase of the Na_v_1.8 mediated current (Liu et al., 2010). Furthermore, using a site-directed mutagenesis approach, the authors proposed that the forward trafficking effects of Na_v_1.8 could be due to the phosphorylation of an identified ER retention signal.

Na_v_1.9 is also increased by inflammatory mediators (Maingret et al., 2008), such as PGE_2_. This mechanism is dependent on G-protein activation (Rush and Waxman, 2004) and GTPγS (Vanoye et al., 2013).

### Protein Kinase B

The PKB (also referred to as Akt) is well-known for its role in neuronal plasticity in the brain (Sanna et al., 2002), but little is known about its role in the pain field. There is evidence, however, that PKB is activated in sensory neurons in animal models of neuropathic pain (Xu et al., 2007a; Shi et al., 2009) and inflammatory pain (Zhuang et al., 2004; Sun et al., 2006). Furthermore, intrathecal injection of a PKB inhibitor attenuated formalin and carrageenan-induced hypersensitivity (Xu et al., 2011). The authors demonstrated that this effect was due to mTOR signaling in the spinal cord, but the intrathecal injection mode of delivery cannot rule out an additional effect in the peripheral nervous system. A recent study showed that PKB activation in peripheral sensory neurons was necessary for the inflammatory-induced increased expression of both Na_v_1.7 and Na_v_1.8 (Liang et al., 2013) since blocking this kinase reversed the upregulation of both isoforms. The previous study did not demonstrate that PKB could directly phosphorylate Na_v_s, nor has any other study to our knowledge. However, ASICS, another important ion channel implicated in pain processing, can be directly phosphorylated by PKB, leading to increased trafficking and enhanced expression of ASICs at the membrane (Duan et al., 2012). Further investigations are required to unravel the role of PKB in the phosphorylation of Na_v_ channels in pathological pain.

### Protein Kinase C

The implication of PKC activation in nociceptive neurons has been extensively studied. Activation of PKC by phorbol esters demonstrated an *in vivo* implication of this pathway in peripheral sensitization (Rang and Ritchie, 1988; Schepelmann et al., 1993; Souza et al., 2002). PKC inhibition decreased hyperalgesia in a model of diabetic neuropathy. Together with PGE_2_, bradykinins are also able to activate PKC pathways (Cesare and McNaughton, 1996; Ferreira et al., 2005, 2008). PKC is a serine/threonine kinase, which has at least 12 different isoforms (Way et al., 2000) that can be classified into different groups (Battaini, 2001). PKC𝜀 is one of the isoforms shown to be necessary for the development of hypersensitivity in animal models of peripheral chronic pain (Khasar et al., 1999; Aley et al., 2000; Dina et al., 2000, 2001). Inflammatory molecules enhance the translocation of PKC**𝜀** to the membrane, where it contributes to peripheral sensitization (Khasar et al., 1999; Hucho and Levine, 2007; Zhang et al., 2007). Other PKC isoforms have also been implicated in chronic pain, but focus has been on their expression in the spinal cord.

As for PKA, the phosphorylation of Na_v_1.2 by PKC (Costa and Catterall, 1984b) could be responsible for a reduction of up to 80% of the current when expressed in *Xenopus* oocytes, with a concomitant slowing of its inactivation (Numann et al., 1991). This reduction is due, at least in part, to a positive shift in the voltage-dependence of activation (Dascal and Lotan, 1991). Similar findings on the total sodium current have been observed in rat brain neurons (Numann et al., 1991; Cantrell et al., 1996). Other studies in *Xenopus* oocytes have shown that PKC downregulates the skeletal muscle sodium channel Na_v_1.4 (Bendahhou et al., 1995), the cardiac channel Nav1.5 (Murray et al., 1997), as well as the two pain specialized isoforms Na_v_1.7 and Na_v_1.8 (Vijayaragavan et al., 2004a). It was initially hypothesized that PKC robustly downregulates Na_v_s across species since a reduction in the Na_v_1.5 current was also observed in myocytes and CHO cells (Qu et al., 1994), and a reduction in the total sodium current was observed in neuroblastoma cells (Renganathan et al., 1995), hippocampal neurons (Cantrell et al., 1996), and cortical neurons (Mittmann and Alzheimer, 1998).

As with PKA activation, the decrease of the Na_v_1.7 current mediated by PKC activation (Vijayaragavan et al., 2004a) is discrepant with the increased excitability observed in pathological pain. Again, the cellular background studied could explain these discrepancies. Furthermore, a study reported that blocking PKC phosphorylation was concomitant with a decrease in Na_v_1.7 protein expression upon continuous opioid administration in a diabetic painful neuropathic model (Chattopadhyay et al., 2008). In addition, a recent study also showed that PMA activation of PKC pathways led to an increase of Na_v_1.7 resurgent currents in HEK cells (Tan et al., 2014), currents which have been implicated in pathological pain (Jarecki et al., 2010).

Gold et al. (1996, 1998) observed that the activation of PKC increased the TTX-resistant current in nociceptive neurons. This was later confirmed by another group (Ikeda et al., 2005). A later study determined which PKC isoform and which of the two TTX-resistant isoforms were responsible for the increased current, implicating PKC𝜀 and Na_v_1.8 (Cang et al., 2009). The PKC𝜀 mediated upregulation of Na_v_1.8 was confirmed in another study, demonstrating the causative link to hyperalgesia (Wu et al., 2012). Since PGE_2_ can activate the PKC pathway and increase the Na_v_1.9 current in nociceptive neurons (Rush and Waxman, 2004), it is likely that both Na_v_1.8 and Na_v_1.9 are implicated. Na_v_1.8 and Na_v_1.9 are well-identified sodium channels whose expression and function are increased upon pathological pain-driven PKC activation. The role of Na_v_1.7, however, remains to be fully elucidated. Since PKA and PKC pathways converge and co-regulate Na_v_1.2 function (Chahine et al., 2005), similar mechanisms might also account for the effects on Nav1.7, Na_v_1.8 (Gold et al., 1998) and Na_v_1.9.

### MAPK Pathway

Mitogen-activated protein kinases are another family of kinases that play an important role in mammalian cell signaling. There are three major members in the MAPK family: ERK, p38 and c-JUN. Each member activates a specific intracellular pathway (Widmann et al., 1999). They regulate various cellular activities and have been implicated in numerous human diseases, including tissue injury (Kim and Choi, 2010). Since they are activated by proinflammatory cytokines (Ji et al., 2009), they have been shown to be important in pathological pain (Obata and Noguchi, 2004).

In transected axons of experimental neuromas, Na_v_1.7 accumulates with ERK1/2 at the site of injury (Persson et al., 2011). Since ERK1/2 was shown to phosphorylate Na_v_1.7, altering its biophysical properties and rendering it easier to open in response to stimuli (Stamboulian et al., 2010), it is hypothesized that a Na_v_1.7 and ERK1/2 co-accumulation would increase nociceptive neuronal firing. Another study proposed that IL-6 application enhances the excitability of trigeminal ganglion neurons via ERK-mediated phosphorylation of Na_v_1.7, a mechanism involved in the development of migraine-related pain behavior (Yan et al., 2012).

Another kinase of the MAPK, p38, was reported to be increased in animal models of neuropathic pain (Obata and Noguchi, 2004; Xu et al., 2007b), leading to the upregulation of TTX-resistant sodium channels in sensory neurons (Jin and Gereau, 2006). A study recently unraveled the role of TNF-α in activating p38, resulting in the modification of the slow inactivation and voltage dependence of activation of Na_v_1.8/Na_v_1.9. This, in turn, increases the TTX-resistant inward current and enhances nociceptive hyperexcitability (Gudes et al., 2015). Another study showed that p38 phosphorylates Na_v_1.8, increasing the trafficking of this channel at the membrane of DRG neurons (Hudmon et al., 2008).

Similar to the previous studies investigating MAPK activation in animal models of chronic pain and those focusing on the regulation of Na_v_1.7 and Na_v_1.8 by kinases *in vitro*, ERK1/2 and p38 are increased along with the Na_v_1.7 and Na_v_1.8 isoforms in painful human neuromas (Black et al., 2008), where they likely also contribute to neuronal hyperexcitability.

### CamKII

Calmodulin (CaM) is a small calcium-binding protein that senses Ca^2+^ changes and drives cellular responses to rapid changes in intracellular calcium concentration. It is also known to be involved in regulating Ca^2+^-dependent neuronal plasticity (Solà et al., 2001).

The C-terminus of Na_v_s contains a CaM-binding domain, known as the IQ motif (Mori et al., 2000), which is a recognizable site for these calcium-sensing proteins. Even though the functional meaning of such binding remains unknown, there are a few studies reporting the regulation of Na_v_ currents by CaM. Despite the fact that all Na_v_s possess a conserved IQ motif, the regulatory effect of CaM is isoform specific, as exemplified by the more potent regulation of Na_v_1.4 as compared to Na_v_1.5 by this protein (Deschênes et al., 2002; Young and Caldwell, 2005; Biswas et al., 2008; Ben-Johny et al., 2014). CaM also regulates Na_v_1.6, which is expressed in nociceptive neurons (Herzog et al., 2003). Interestingly, Na_v_1.7 is also bound by CaM (Herzog et al., 2003), but with a lower affinity than Na_v_1.6.

CaM Ca^2+^-dependent activation leads to the activation of many signaling molecules, one being the Ca^2+^/CaM-dependent serine/threonine kinase (CamK) (Nelson and Chazin, 1998). CamKII is expressed in nociceptive neurons and is involved in pain transmission (Hiruma et al., 1999; Brüggemann et al., 2000). It was proposed that CamKII is responsible for the transition from acute to chronic pain, a process involving PKC𝜀 activation (Ferrari et al., 2013) and links these two kinase pathways. Further evidence supporting a role for CamKII in neuropathic pain was demonstrated by using a CamKII inhibitor that reversed mechanical allodynia in animal models of both neuropathic pain (Chen et al., 2009) and inflammatory pain (Luo et al., 2008a).

Since the CaM binding effect on Na_v_s regulation is thought to be due to the recruitment and subsequent phosphorylation of Na_v_s by CamKII (Deschênes et al., 2002; Maltsev et al., 2008), it is likely that the activation of this kinase in pathological pain states is partially responsible for hyperexcitability through the regulation of Na_v_. This hypothesis remains to be confirmed.

## Glycosylation

Glycosylation is another important PTM that affects sodium channel function and expression. Glycosylation is a crucial step for protein biosynthesis and folding, but it is also involved in cell signaling, cell-cell adhesion, protection against proteolysis, and cellular development and immunity (Moremen et al., 2012). Na_v_ α-subunits undergo important glycosylation steps in the endoplasmic reticulum and Golgi apparatus (Waechter et al., 1983; Schmidt and Catterall, 1987), a process involving the sequential addition of *N*-acetylglucosamines capped by sialic acid residues, and the further addition of diverse oligosaccharide chains. Glycosylation can account for 5% (Cohen and Barchi, 1993) to 30% of the α-subunit’s molecular weight (Messner and Catterall, 1985), depending on the isoform, with an estimated stoichiometry of around 100 sialic acid molecules per channel (James and Agnew, 1987). Glycosylation is known to influence Na_v_ gating properties (Recio-Pinto et al., 1990; Bennett et al., 1997; Zhang et al., 1999; Tyrrell et al., 2001) by interfering with the electric field near the gating sensors (Bennett et al., 1997; Cronin et al., 2004). It was proposed that extracellular sialic acid residues, which are negatively charged at physiological pH, influence the sensitivity of the voltage sensor domains to the transmembrane electrical potential difference (Ednie and Bennett, 2011).

Only a few studies have investigated glycosylation of Na_v_s in the peripheral sensory nervous system. One reported that Na_v_1.9 is subject to important developmentally regulated glycosylation. This isoform is found in two different heavily glycosylated forms in neonatal rats, which have different gating properties as compared to the less glycosylated form of Na_v_1.9 in adult tissue (Tyrrell et al., 2001). Another study reported that Na_v_1.7 is found in at least two different glycosylated forms in HEK293 cells: a heavily functional glycosylated form and a core-glycosylated immature form (Laedermann et al., 2013a). The same authors later reported that a third intermediate glycosylated form is also present in HEK293 cells (Laedermann et al., 2013b). Inhibition of glycosylation in *Xenopus* oocytes by tunicamycin also altered Na_v_1.3 gating properties (Xu et al., 2008). Whether these different patterns of α-subunit glycosylation lead to modification of Na_v_ function has yet to be investigated in peripheral chronic pain syndromes.

## Ubiquitylation

Ubiquitylation is another well-known PTM that negatively regulates the cell surface expression of many different plasma membrane proteins (Staub and Rotin, 2006). Ubiquitylated proteins that are internalized through this pathway are either degraded or recycled (Shih et al., 2000; Abriel and Staub, 2005; Ciechanover, 2005). Ubiquitin is a small and highly conserved polypeptide of 76 amino acids that is covalently attached to the lysine residues of the targeted protein. Three enzymatic successive steps are required to ubiquitylate a protein (Pickart, 2001): (1) ubiquitin is first activated by a ubiquitin-activating enzyme (E1) in an ATP-dependent manner, (2) ubiquitin is then transferred to a ubiquitin-conjugating enzyme (E2) via a thioester bond, (3) this complex further interacts with an ubiquitin-protein ligase (E3) that eventually ubiquitylates the substrate protein.

The first and probably best-described protein that undergoes ubiquitylation is ENaC (Abriel et al., 1999; Rossier et al., 2002). The ENaC subunit possesses a PY motif. Mutating this motif is sufficient to generate a hypertensive phenotype, known as Liddle’s Syndrome (Schild et al., 1996), which involves increased ENaC function (Firsov et al., 1996). Nedd4 and Nedd4-2 proteins where shown to bind to ENaC’s PY motif on large members of the E3 ubiquitin ligase family (Joazeiro and Weissman, 2000; Metzger et al., 2012), leading to its internalization (Abriel et al., 1999; Henry et al., 2003). This process is impaired in Liddle’s syndrome.

Most Na_v_s possess a PY motif at their α-subunit C-terminal, making them potential substrates for Nedd4-2 dependent ubiquitylation (Abriel et al., 2000; Laedermann et al., 2014a). The first sodium channel isoform described to be regulated by Nedd4-2 was Na_v_1.5. Regulation was demonstrated in both *cell expression systems* and cardiac tissue (van Bemmelen et al., 2004). Na_v_1.7 and Na_v_1.8 also possess a PY motif and were shown to be negatively regulated by Nedd4-2 in both *Xenopus* oocytes (Fotia et al., 2004) and HEK293 cells (Laedermann et al., 2013a). The functional significance of Na_v_ regulation by Nedd4-2 was previously demonstrated in chronic pain syndromes. In an animal model of neuropathic pain, ubiquitin ligase expression was robustly reduced in both mice (Laedermann et al., 2013a) and rats (Cachemaille et al., 2012). Na_v_1.7 and Na_v_1.8 expression were increased as a consequence of Nedd4-2 downregulation. The causal link between Nedd4-2 downregulation and Na_v_1.7/Na_v_1.8 upregulation was demonstrated using both tissue specific knockout and viral overexpression of Nedd4-2, leading to hyper- and hypo- pain sensing phenotypes, respectively (Laedermann et al., 2013a). The reduction of Nedd4-2 might also increase membrane expression of other Na_v_s, with the exception of Na_v_1.9 which lacks the PY motif, (Abriel et al., 2000), and other ion channels expressed in sensory neurons that are also substrates for this ubiquitin ligase (Bongiorno et al., 2011). Na_v_1.6 was also regulated by Nedd4-2 in mouse hippocampal neurons, a process dependent on the concomitant p38-mediated phosphorylation of this sodium channel isoform (Gasser et al., 2010). Whether Nedd4-2 downregulation in neuropathic pain is due to the inflammatory soup or to another mechanism remains to be investigated.

## Methylglyoxal

A recent study unraveled a new mechanism accounting for painful peripheral neuropathy in diabetes. The authors showed that the concentration of methylglyoxal, an endogenous degradation product of excessive glycolysis (Thornalley, 2005), is increased in patients suffering from diabetes. Since peripheral nerves have low levels of enzymes that metabolize methylglyoxal (Bierhaus and Nawroth, 2009), its accumulation in the sensory system was proposed to account for pain hypersensitivity. The authors reported that the methylglyoxal effect on excitability was through its binding to Na_v_1.8 within the DIII-DIV linker on an arginine residue, which reduced channel inactivation (Bierhaus et al., 2012), leaving the channel in an excitable state. In sciatic nerve biopsies isolated from patients with diabetes and from those who had amputations due to peripheral artery disease, the authors observed an increase of Na_v_1.8 modification by methylglyoxal when compared to controls. It was proposed that this PTM was specific to Na_v_1.8 in regards to sodium channel regulation, but the authors demonstrated that methylglyoxal can also have an effect on pain pathways by depolarizing nociceptive neurons, and increasing GCRP release and COX-2 expression.

## Post-Translational Modification Of Na_v_ Protein Partners

Many protein partners are known to interact with typical pain isoforms and undergo PTM. Evidence is lacking which demonstrates a direct effect between protein partners’ PTMs and concomitant modification of Na_v_ expression or function. In the following chapter, we will discuss the potential partners that undergo PTMs that are known to regulate Na_v_1.7, Na_v_1.8, and Na_v_1.9.

### β–Subunits

β–subunits are important regulators of Na_v_s. They are implicated in neuropathic pain and are subject to PTMs. β-subunits regulate α-subunit gating properties by direct steric interactions that interfere with the voltage-sensor (Zimmer and Benndorf, 2002). Even though the effects of the different β-subunits on biophysical properties give rise to conflicting results (Nuss et al., 1995; Sangameswaran et al., 1997; Smith and Goldin, 1998; Morgan et al., 2000; Fahmi et al., 2001; Vijayaragavan et al., 2001, 2004b; Zimmer and Benndorf, 2002), partially due to the different cell types used, the literature clearly demonstrates that β-subunits regulate Na_v_ gating. β-subunits can also affect the Na_v_ current in an *ex vivo* nociceptive neuron primary culture, as highlighted by the decreased *I*_Na_ current recorded from *SCN1B* and *SCN2B* knockout animals (Lopez-Santiago et al., 2006, 2011). *SCNB* knockout animals also demonstrated abnormal pain sensing, confirming that modulating Na_v_ function ultimately modulates pain signaling (Pertin et al., 2005; Lopez-Santiago et al., 2006, 2011). Moreover, β1-, β2-, and β3-subunit expression levels are increased in different animal models of pathological pain (Shah et al., 2000; Coward et al., 2001b; Pertin et al., 2005), highlighting their potential implication in modulating cellular excitability. Auxiliary β-subunits are themselves substrates for glycosylation (Isom et al., 1992), which ultimately modulates α-subunit function (Johnson et al., 2004; Johnson and Bennett, 2006). β-subunits can also be phosphorylated, i.e., the phosphorylation of Tyr181 is necessary for the interaction with ankyrin, another Na_v_ protein partner (Malhotra et al., 2002). Whether altered glycosylation or phosphorylation of the β-subunits could be implicated in pathological pain has, at least to our knowledge, never been investigated.

### Nedd4-2

Nedd4-2, a potent regulator of Na_v_s in the sensory nervous system, also undergoes several PTMs, particularly by kinases which alter its function. Most of these pathways have been investigated in the Nedd4-2 regulation of ENaC (Snyder, 2009). Whether a similar action also occurs on the sodium channels in the sensory system remains to be determined. Pathways that lead to Nedd4-2 PTMs are the same pathways as those activated in chronic pain syndromes. For instance, phosphorylation of Nedd4-2 by PKA was demonstrated to decrease Nedd4-2 ubiquitylating efficiency (Snyder et al., 2004). In addition to its direct effect on Na_v_s, it is probable that PKA activation also indirectly leads to an increase of Na_v_ channels at the membrane by impeding the downregulatory role of Nedd4-2. It has been well documented that cytokine signaling leads to NF-κB activation in inflammatory processes. These signals first activate the IκB kinase [an inhibitor of nuclear factor κB (NF-κB)] (IKK) via phosphorylation, which then phosphorylates and inactivates IκB inhibitory proteins. IKKβ, a subunit of IKK that is expressed in unmyelinated fibers, then binds and phosphorylates Nedd4-2 (Edinger et al., 2009). Finally, NGF, another inflammatory mediator (Leung and Cahill, 2010) also known to positively regulate Na_v_s mRNA expression (Toledo-Aral et al., 1997; Fjell et al., 1999), triggers a cascade after binding to the TrkA receptor, which ultimately leads to Nedd4-2 phosphorylation (Arévalo et al., 2006). Pro-inflammatory molecules likely negatively regulate Nedd4-2 activity in nociceptive neurons following inflammation, leading to an increase in Na_v_ expression at the membrane.

### Collapsin Response Mediator Protein 2 (CRMP2)

Collapsin response mediator protein 2 (CRMP2) is a protein initially identified to be important for axonal outgrowth (Inagaki et al., 2001). It is now known to be important for modulating ion channel trafficking (Bretin et al., 2006; Chi et al., 2009). CRMP2 is the secondary target of the anti-epileptic drug lacosamide (Errington et al., 2006, 2008) and can directly bind to Na_v_s (Wang et al., 2010), modulating the channel’s slow inactivation. In a recent study carried out in both HEK293 cells and sensory neurons, it was demonstrated that CRMP2 can be SUMOylated, which affects Na_v_1.7 trafficking (Dustrude et al., 2013). Whether SUMOylation of CRMP2 plays a role in pathological pain via the alteration of Na_v_1.7 trafficking remains to be investigated.

There are many other well described proteins, i.e., contactin (Ranscht, 1988; Kazarinova-Noyes et al., 2001), ankyrin (Malhotra et al., 2000), spectrin (Bennett and Baines, 2001), dystrophin, and syntrophin (Gee et al., 1998; Abriel and Kass, 2005) among others (Shao et al., 2009), that interact with Na_v_s and modulate their function and cell surface expression. Kinases are themselves substrates for PTMs that can modify their function in important ways. All of these proteins are subject to PTMs, which may impact the control of Na_v_ function and expression in peripheral chronic pain syndromes.

## Ptms Modulate Na_v_ Transcription

In studies carried out in adrenal chromaffin cells, which express relatively high levels of Na_v_1.7 (Klugbauer et al., 1995; Toledo-Aral et al., 1995, 1997; Goldin, 2001; Wada et al., 2004, 2008), a role for PTMs in controlling Na_v_ transcription was proposed. ERK kinases were shown to positively regulate Na_v_1.7 expression at the membrane by modulating their mRNA stability (Yanagita et al., 2003). Conversely, PKC𝜀 kinase activation destabilized Na_v_1.7 mRNA, contributing to Na_v_1.7 negative regulation of the steady-state levels at the plasma membrane (Yanagita et al., 1996). Whether similar mechanisms occur in sensory neurons has not been reported.

## Discussion

Na_v_s expression and function are dysregulated in peripheral inflammatory pain, nerve injury induced neuropathic pain, metabolic, infectious, toxic or inherited painful neuropathies. Peripheral mechanisms for initiation and maintenance of pain is partially due to the release of inflammatory molecules that trigger different signaling cascades and leads to the activation of enzymes with diverse functions. In turn, these enzymes will post-translationally modify Na_v_ function and expression, ultimately impacting nociceptive neuronal hyperexcitability and pain. Most knowledge of such mechanisms comes from the kinase field, but since Na_v_s are large proteins composed of over 2000 amino acids, they possess a plethora of domains that could be subject to PTMs. It is likely that PTM’s contribution to chronic pain syndromes are still under-estimated and should be further investigated. In this review, we not only discussed proven Na_v_ PTMs implications in pathological pain but also other potential relevant PTMs, which will eventually open new avenues for a deeper understanding of chronic pain syndromes. Together with the development of more efficient and specific pharmacological agonists and antagonists targeting PTM effectors, new genome engineering tools will facilitate the generation of knock-in mice with mutations on PTM sites or on the PTM effectors themselves. This will hopefully help confirm and identify new pathways that regulate Na_v_ function and expression.

The pharmaceutical industry is attempting to develop highly selective drugs that block Na_v_1.7, Na_v_1.8, or Na_v_1.9, but with unfortunately little progress. This is partially due to the fact that Na_v_s are well-conserved proteins, and non-specific blockers are bound to have many dramatic side effects. Rather than targeting and blocking the sodium channels themselves, regulating the function or expression of sodium channels, which may have more subtle effects on excitability, might prove to be an interesting alternative to treat chronic pain. From this perspective, more effort should be focused on targeting PTM pathways, as exemplified by the anti-TNF-α drug, which is promising and still in expansion (Leung and Cahill, 2010). Pharmacological targeting of the MAPK pathway has already been shown to have an analgesic effect (Tong et al., 2012), and many other kinases are currently in pre-clinical or clinical studies (Ji et al., 2007). Other promising avenues include the use of scavengers of methylglyoxal (Bierhaus et al., 2012) or the use of gene therapy to restore Nedd4-2 ubiquitylating function, which have proven to be efficient in relieving pain in animal models of chronic pain. Altogether, it is important to further characterize the known PTM effects on Na_v_s and to identify new PTMs in order to gain insight into the development of pathological pain.

## Conflict of Interest Statement

The authors declare that the research was conducted in the absence of any commercial or financial relationships that could be construed as a potential conflict of interest.
